# Shikonin suppresses proliferation of osteosarcoma cells by inducing ferroptosis through promoting Nrf2 ubiquitination and inhibiting the xCT/GPX4 regulatory axis

**DOI:** 10.3389/fphar.2024.1490759

**Published:** 2024-12-05

**Authors:** Rui Huang, Dawei Chu, Jiandang Shi, Ruiqing Xu, Kun Wang

**Affiliations:** ^1^ General Hospital of Ningxia Medical University, The First School of Clinical Medicine, Yinchuan, Ningxia, China; ^2^ Department of Orthopedic Surgery, General Hospital of Ningxia Medical University, Yinchuan, Ningxia, China; ^3^ Department of Orthopedic Surgery, Honghui Hospital, Xi’an Jiaotong University, Xi’an, China

**Keywords:** shikonin, osteosarcoma, ferroptosis, Nrf2, XCT, GPx4

## Abstract

Osteosarcoma (OS) is a prevalent primary malignant bone tumor which lacks effective therapeutic interventions. Ferroptosis is a new form of programmed cell death characterized by iron-dependent accumulation of lethal lipid oxidation, which provides a potential alternative intervene for the OS treatment. Shikonin is the major bioactive component extracted from the roots of lithospermum erythrorhizon which is also known as “Zicao” in traditional Chinese medicine, has been proved to have exhibits remarkable anti-tumor properties in several cancers. However, whether ferroptosis participated in the shikonin mediated anti-OS activity still remains to be clarified. Herein, we provide evidence that shikonin possesses the capability to induce the ferroptosis, and elucidate the underlying mechanisms in the treatment of OS. In the present study, it was found that shikonin significantly suppressed OS cells proliferation and blocked the cell cycle progression *in vitro*. Subsequent results revealed that shikonin could trigger ferroptosis in OS cells by promoting the Fe^2+^ accumulation, reactive oxygen species and lipid peroxidation formation, malondialdehyde production and mitochondrial damage. Further study showed that the effects of OS cell proliferation and death caused by shikonin could be successfully reversed by ferroptosis inhibitor ferrostatin-1, indicating that ferroptosis participated in the shikonin mediated anti-OS activity. Mechanistically, shikonin physically interacted with Nrf2, a critical regulator of ferroptosis, and influenced Nrf2 stability via inducing ubiquitin degradation, which suppressed the expression of Nrf2 downstream targets xCT and GPX4, and led to stimulating ferroptosis. Collectively, our findings indicated that shikonin induced OS cells ferroptosis through Nrf2/xCT/GPX4 regulatory axis, which might shed light on exploiting shikonin as a promising candidate for the future OS therapy.

## 1 Introduction

Osteosarcoma (OS) is the most prevalent primary bone malignancy in children and adolescents, originating from mesenchymal tissues, with 3.4 cases per million people worldwide (Liu W. et al., 2022; Li H. B. et al., 2022). There is a high tendency to metastatic spread of patients with OS (Tang et al., 2019). In general, the mechanism of OS is complex, involving a variety of biomolecular and genetic signaling pathways. The tumor microenvironment and several major signaling pathways including PI3K, JAK/STAT, Wnt/β-catenin, NOTCH, Ras, TGF-β, MAPK/AKT/mTOR, RANK/RANKL, and NF-κB plays a key role in the proliferation, invasion and metastasis of OS (Li et al., 2023). Currently, the treatment of OS mainly adopts a comprehensive treatment based on surgery, combined with radiotherapy, chemotherapy as well as targeted therapy and other treatments (Wakamatsu et al., 2019). Despite significant advances in early diagnosis and treatment of OS in recent years, severe systemic toxic side effects often prevent chemotherapy from being completed.

Shikonin is the major bioactive component extracted from the roots of lithospermum erythrorhizon which is also known as “Zicao” in traditional Chinese medicine (TCM) (Guo et al., 2019). Recent studies have shown that shikonin exhibits various bioactivities associated with the treatment of cancer, inflammation, and wound healing. It was reported that shikonin suppressed tumor growth, induced apoptosis and inhibited metastasis in various cancers (Boulos et al., 2019), suggesting it may be an excellent potential of clinical use and potential anti-cancer candidate.

Ferroptosis is a novel form of regulated cell death (RCD) that characterized by iron dependent and intracellular oxidative accumulation (Chen et al., 2021). Compared with normal cells, high concentration of unstable iron pool is an important substance and metabolic basis for abnormal proliferation, drug resistance and maintenance of tumor stem cells (Cheng et al., 2022), and ferroptosis may be proposed as an alternative intervene for cancer therapy. Currently, there are several Food and Drug Administration (FDA) approved drugs that target ferroptosis, such as erastin and deferoxamine mesylate. However, their unsatisfactory chemical properties, severe adverse reactions and poor tumor targeting limit its clinical application. It is surprising that several natural chemicals could induce ferroptosis and suppress tumor growth such as red ginseng polysaccharide (Zhai et al., 2022), agrimonolide (Liu Y. et al., 2022), tiliroside (Yang et al., 2023) and ginsenoside Rh3 (Wu Y. et al., 2023). Shikonin was also reported to exhibit the anti-tumor effects via inducing ferroptosis in multiple myeloma (Li W. et al., 2022). Therefore, we wondered whether ferroptosis was participated in the shikonin mediated anti-OS activities.

In the present study, shikonin for the first time demonstrated to suppress OS cell growth *in vitro* through inducing ferroptosis, and the regulatory axis Nrf2/xCT/GPX4 was involved in this process. The findings could provide a new idea for further improving the prognosis for patients with OS, and provide a theoretical basis and solid foundation for the clinical application of shikonin.

## 2 Materials and methods

### 2.1 Cell lines and cell culture

The OS cell lines MG63 and143B were cultured in Dulbecco’s modified Eagle’s medium (DMEM, Viva cell) supplemented with 10% fetal bovine serum (FBS, Viva cell). The OS cell lines MG63 and143B were originally obtained from the American Type Culture Collection (ATCC) and were maintained at 37°C in a humidified atmosphere consisting of 5% CO_2_.

### 2.2 Reagents and antibodies

Shikonin, ferrostatin-1 (Fer-1), MG132 and Cycloheximide (CHX) were purchased from MedChemExpress (MCE, State of New Jersey, United States), and the drug was dissolved in DMSO and stored in −20°C for usage. Primary antibody used in the experiments included GAPDH (1:2000; Proteintech, Wuhan, China), β-actin (1:2000; Proteintech, Wuhan, China), PCNA (1:2000; Proteintech, Wuhan, China), GPX4 (1:1000; Proteintech, Wuhan, China), xCT (1:1000; Proteintech, Wuhan, China), Nrf2 (1:1000; Proteintech, Wuhan, China and Affinity, Jiangsu, China) and Keap1 (1:1000; Proteintech, Wuhan, China).

### 2.3 Cell transfection

For evaluating the overexpression of Nrf2, an overexpression plasmid was obtained from Sangon Biotech (Sangon, Shanghai, China). As the manufacturer’s recommended protocol, RNA was transfected using Lipofectamine 3,000 reagent (Thermo Fisher Scientific) for 48 h.

### 2.4 Cell viability assay

MG63 and143B cells were seeded at a density of 8×10^3^ cells/well in a 96-well plate. After being cultured overnight, cells were treated with different concentrations of shikonin (0, 0.25, 0.5, 1, 2, 4, 6, 8 and 10 uM) for 24 or 48 h. The optimal experimental dose and treatment time of the drug were determined based on concentration and time effects of subsequent cell experiments, with 3 wells per group. Cells were treated accordingly, and 10 µL of CCK-8 solution (Abbkine Scientific Co., China) was added to each well. Cells were incubated in an incubator for 2 h. After incubation, the optical density (OD) values were measured at 450 nm using an enzyme immunoassay analyzer, and the cell proliferation inhibition rate was calculated (Cell viability (%) = [(Abs of experimental group - Abs of blank group)/(Abs of control group - Abs of blank group)] × 100%). The half maximal inhibitory concentration (IC_50_) values were calculated using GraphPad Prism 8.0 software (GraphPad Software Inc., San Diego, CA, United States).

### 2.5 Giemsa staining

Giemsa staining was used to observe the effect of shikonin on MG63 and 143B cell morphology. Cell morphology was evaluated using an optical microscope (Olympus Corporation, Tokyo, Japan).

### 2.6 Colony formation assay

MG63 and 143B cells at the logarithmic growth phase were inoculated into 6-well plates with 500 cells/well in a single-cell suspension. After 48 h, MG63 and 143B cells were treated with shikonin or vehicle control to evaluate the proliferation potential of the cell lines in presence and absence of shikonin, with 3 wells per group. The medium containing different concentrations of drugs was changed every 3 days, and the medium was discarded after the colony formation (clones with >50 cells). Cells were fixed by 4% PFA and stained by 0.1% crystal violet (Solarbio, China). The results were processed using ImageJ software.

### 2.7 Cell cycle analysis

MG63 and 143B cells were plated in a 6-well plate, at a concentration of 2 × 10^5^cells/well. When the cell density reached about 80%, intervention was performed according to the protocol. The MG63 and 143B cells were intervened with different concentrations of shikonin (0, 075, 1.5, 3 uM) for 24 h and were harvested by trypsinization, washed in cold PBS twice, and placed in 70% ethanol overnight at 4°C. After that, cells were incubated in a solution with DNA-binding dye PI, RNase A (Key GEN Biotech, Nanjing, China) for 30 min at 37°C in the dark. Finally, red fluorescence from 488 nm laser-excited PI in every cell was analyzed by flow cytometer. ModFit software was used to determine the percentage of cells in G0/G1, S, and G2/M phases.

### 2.8 Transmission electron microscopy

MG63 and 143B cells were treated with or without 1.5 uM shikonin for 24 h, then they were scraped off and placed in a centrifuge tube. After centrifugation, the samples were treated according to the manufacturer’s protocol, and observed under a transmission electron microscope (Olympus Corporation, Tokyo, Japan).

### 2.9 Flow cytometric analysis of detecting reactive oxygen species (ROS), lipid peroxidation and Fe^2+^

Flow cytometry was applied to detect ROS, lipid peroxidation and Fe^2+^. Briefly, shikonin-treated MG63 and 143B cells were centrifuged and washed thrice with PBS. The cells were stained with 10 uM DCFH-DA (Invitrogen, Carlsbad, CA, United States) for 30 min at 37°C in the dark. And then washed twice with warm HBSS and harvested by trypsinization. It was then resuspended with PBS and analyzed by flow cytometry using AccuriC6 plus within 20 min. For detection of intracellular lipid peroxidation, C11-BODIPY (Thermo Fisher Scientific, Waltham, United States) was used to detect the level of lipid peroxidation, the fluorescence was examined by flow cytometry. For detection of intracellular Fe^2+^ content, the cells were stained with 1 uM FerroOrange (DOJINDO, Shanghai, China) for 30 min at 37°C in the dark, the fluorescence was monitored by flow cytometry. Collected data were analyzed using Flow Jo 10.7.1 software.

### 2.10 Real-time quantitative PCR (qRT-PCR)

After exposure to 1.5 uM shikonin for 24 h, total RNA was isolated and reverse-transcribed into cDNA by using a cDNA transcription kit. Quantitative RT-PCR was carried out with a real-time fluorescence quantitative RT-PCR detection system (Applied Biosystems, Life Technologies). β-actin was used as loading controls. The primers for genes were listed as follows: GAPDH forward 5’-GTC TCC TCT GAC TTC AAC AGC G-3’, and GAPDH reverse 5’-ACCACC CTG TTG CTG TAG CCA A-3’; Nrf2 forward 5’-CAC ATC CAG TCAGAA ACC AGT GG-3’, and Nrf2 reverse 5’-GGA ATG TCT GCG CCA AAAGCT G-3’.

### 2.11 Measurement of malondialdehyde (MDA) levels

Intracellular concentrations of MDA were detected by an MDA Assay Kit (Jiancheng, Nanjing, China). The detailed measurement was conducted according to the kit instructions.

### 2.12 Measurement of GSH/GSSG ratio

Intracellular concentrations of GSH/GSSG ratio were detected by a GSH/GSSG ratio Assay Kit (Jiancheng, Nanjing, China). The detailed measurement was conducted according to the kit instructions.

### 2.13 Immunofluorescence (IF) analysis

Mitochondrial Fe^2+^ levels were detected using Mito-FerroGreen (Dojindo, Shanghai, China). According to the protocol, the cells were seeded in confocal dishes. After a 24 h intervention with 1.5 uM shikonin co-treated with or without 10 uM Fer-1, the cells were incubated with Mito-FerroGreen for 30 min. The samples were observed under a confocal microscope (Nikon, Japan). Regarding to the mitochondrial ROS detection, the cells were incubated with 5 uM Mito-SOX (Dojindo, Shanghai, China) for 30 min, and observed under a confocal microscope (Nikon, Japan).

### 2.14 Molecular docking

Molecular docking and virtual screening were performed with AutoDock Vina software. The chemical structure of shikonin was obtained from PubChem Compound (https://Pubchem. NCBI. nlm. nih. Gov/). The crystal structure of Nrf2 was retrieved from the Research Collaboratory for Structural Bioinformatics Protein Data Bank (RCSB PDB, http://www.rcsb.org/pdb/). PyMOL2.3.0 software was adopted to remove protein crystalline water, primitive ligands. Molecular docking was performed by AutoDock software, and the molecular docking data were visualized using Discovery Studio2019.

### 2.15 Western blot assay

Cells were lysed after treated with shikonin for 24 h, and total protein was extracted. The protein concentrations were determined using BCA protein assays. Of each sample, 40 ug was added and separated by SDS-PAGE, which was then transferred to a polyvinylidene difluoride (PVDF) membrane. After blocking with 5% nonfat milk for 2 h, the membranes were incubated with the primary antibodies and incubated at 4°C overnight. On the next day, thrice washing with TBST was performed, and the secondary antibodies of the corresponding species were added and incubated at room temperature for 90 min. After thrice washing with TBST, the images were developed in a dark room using ECL chromogenic solution (Abbkine Scientific Co., China) exposed on a gel imaging system (BioRad Laboratories, Hercules, CA, United States ). The results were processed using Image Lab software.

### 2.16 Immunoprecipitation (IP) assay

The cells were treated with shikonin for 24 h and collected, then lysed with the lysis buffer. Then the supernatant after centrifugation was obtained, and incubated with 1 μL antibody and 10 μL protein A/G beans (Bioss, Beijing, China) at 4°C overnight. Then the beans were obtained by centrifugation, and washed 3–4 times. The enriched proteins were suspended by 1× loading buffer and boiled for 15 min, then separated by SDS-PAGE.)

### 2.17 Statistical analysis

All of the *in vitro* experiments were performed at least three times. All of the data are presented as mean ± SD. The differences among the groups were tested by a one-way or two-way analysis of variance (ANOVA). Multiple-comparison tests were applied only when a significant difference was determined by the ANOVA. Values of *p* < 0.05 were deemed to be statistically significant.

## 3 Results

### 3.1 Shikonin inhibited the proliferation of OS cells and induces cell cycle arrest

**FIGURE 1:**
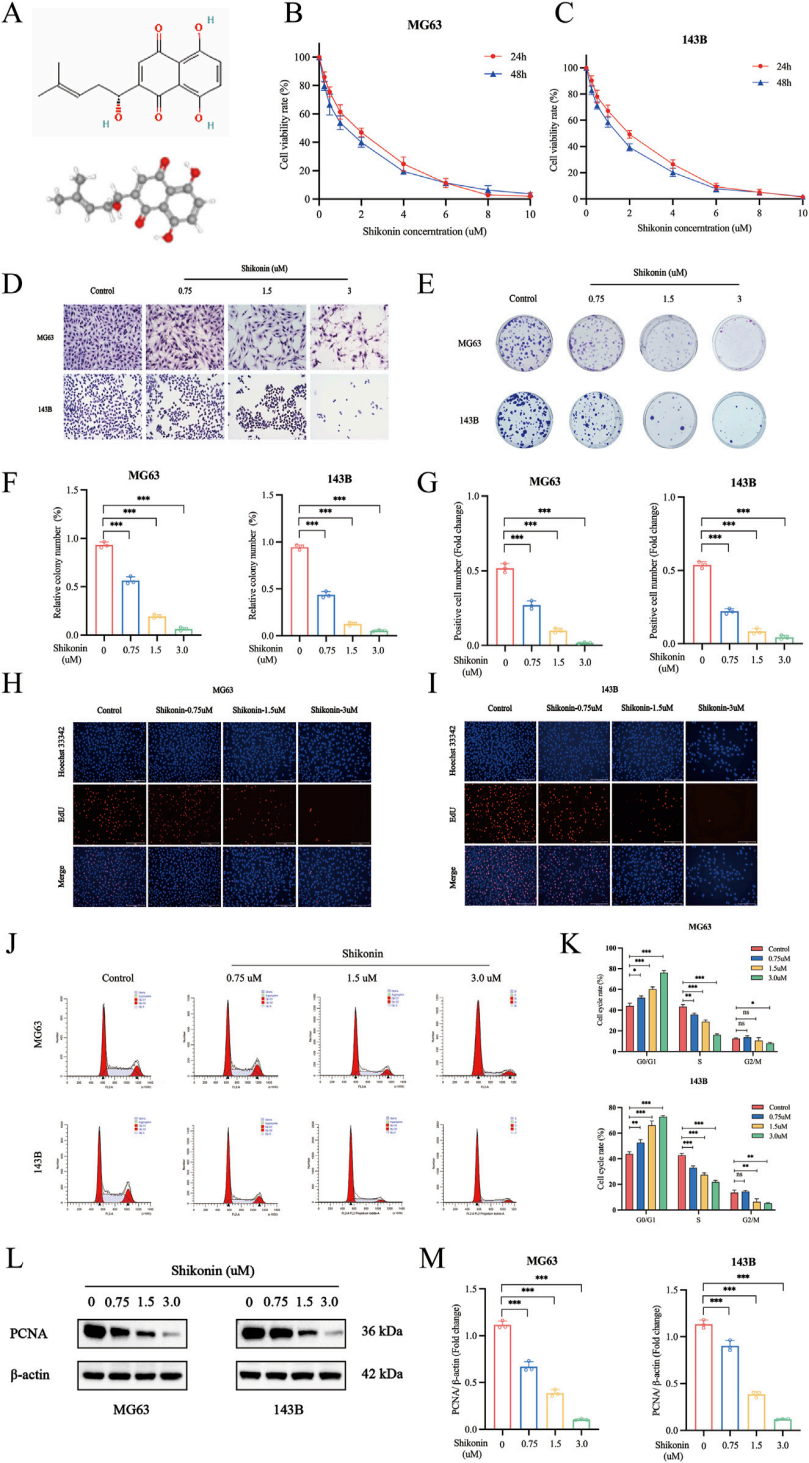
Shikonin inhibited cell proliferation of MG63 and 143B cells. **(A)** The molecular and 3D structure of shikonin. **(B–C)** MG63 and 143B cells were cultured at different concentrations of shikonin (0, 0.25, 0.5, 1, 2, 4, 6, 8 and 10 uM) for 24, 48 h, and then CCK-8 assay was performed to detect the cell proliferation. **(D)** Giemsa staining was used to observe the effect of shikonin on cell morphology. **(E–F)** Colony formation and **(G–I)** EdU Staining were used to observe the impact of shikonin on cell proliferation. **(J, K)** Flow cytometry was used to analyze the effects of shikonin on the cell cycle when cells were treated with or without shikonin for 24 h **(L, M)** Western blot was used to detect the expression of proliferation protein PCNA. *p < 0.05, **p < 0.01, ***p < 0.001. The data were presented as mean ± SD, and analyzed using one-way ANOVA following Tukey’s t-test (n = 3).

Shikonin molecular structure and 3D structure from the PubChem database (https://pubchem.ncbi.nlm.nih.gov) (Figure 1A). The proliferation of different groups of cells was assessed by CCK-8, colony formation assays, EdU staining and Western blot assays. To explore the effect of shikonin on the proliferation of MG63 and 143B cells, we used different concentrations of shikonin (0, 0.25, 0.5, 1, 2, 4, 6, 8 and 10 uM) to intervene MG63 and 143B cells. CCK-8 assay suggested that the cell viability of MG63 and 143B cells decreased both in a dose-dependent and time-dependent after shikonin treatment (*p* < 0.05). The IC_50_ of MG63 and 143B cells treated with shikonin for 24 h was 1.432 uM and 1.638 uM (Figures 1B, C). Therefore, 0.75 uM, 1.5 uM and 3 uM of shikonin concentration were selected for the following experiments. Giemsa staining indicated that cell morphology crumpled and the nucleus was deep staining, decreased OS cells status and viability after shikonin intervention (Figure 1D). The results of colony formation assay were consistent with those of CCK-8 assay (Figures 1E, F). EdU proliferation assays indicated that shikonin inhibits the proliferation of MG63 and 143B (Figures 1G, I) in a dose-dependent manner. The flow cytometry assay showed that shikonin could induce cell cycle arrest at G1→S phase, increased the G1 phase percentage and decreased S, G2/M phase percentage of MG63 and 143B (Figures 1J, K). Furthermore, the proliferation marker PCNA is closely associated with the initiation of cell proliferation. In present study, we found that shikonin significantly downregulated PCNA expression of MG63 and 143B in a dose-dependent manner (Figures 1L, M).

### 3.2 Shikonin triggered the ferroptosis in OS cells

**FIGURE 2:**
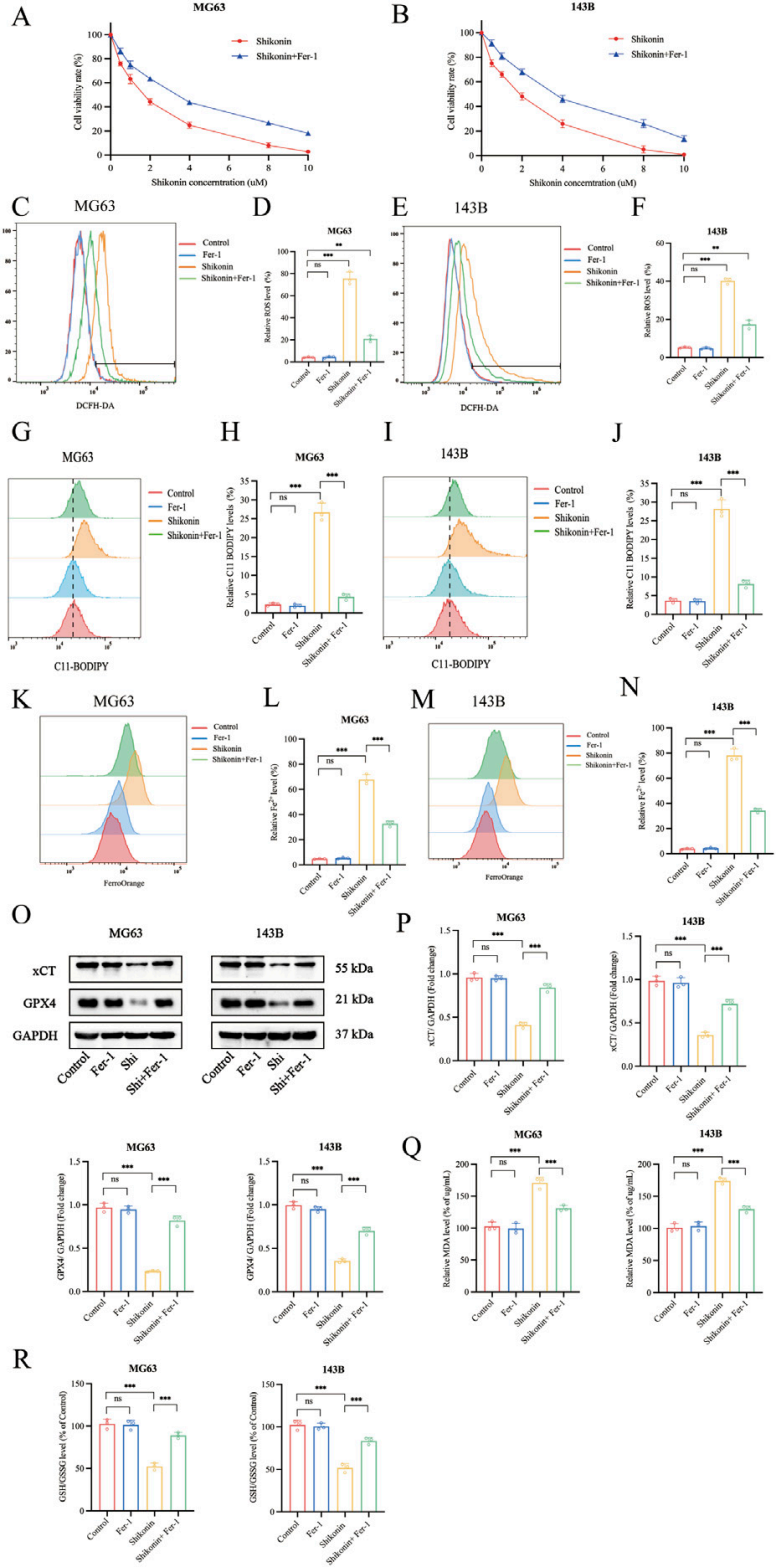
Shikonin triggered the ferroptosis in OS cells. **(A, B)** The MG63 and 143B cells were co-treated with shikonin and Fer-1 after 24 h, and the cell viabilities were examined by CCK-8 assay. **(C–F)** The ROS, **(G–J)** lipid peroxidation and **(K–N)** Fe^2+^ contents were performed by Flow cytometry, followed by quantitative analysis. **(O, P)** Western blot was used to detect the expression of protein xCT and GPX4, and quantification was analyzed. The levels of MDA **(Q)** and GSH/GSSG **(R)** were determined in the shikonin treated OS cells. *p < 0.05, **p < 0.01, ***p < 0.001. The data were presented as mean ± SD, and analyzed using one-way ANOVA following Tukey’s t-test (n = 3).

One major finding of this study is our elucidation of the mechanism underlying the shikonin on anti-proliferation of OS cells. Further results indicated that shikonin could induce OS cells ferroptosis. Ferroptosis was characterized with accumulation of iron, ROS and lipid peroxidation overload, and was negatively regulated by xCT and GPX4 (Wei et al., 2020). MG63 and 143B cells were treated with shikonin (1.5 uM) in the presence or absence of Fer-1 (10 uM) for 24 h, and CCK-8 results showed that Fer-1 could partially reverse shikonin induced reduction in OS cell viability (Figures 2A, B). The level of intracellular ROS, lipid peroxidation and Fe^2+^ was measured separately using a fluorescent probe DCFH-DA, C11-BODIPY589/591 and FerroOrange, and the flow cytometry results showed that shikonin (1.5 uM) treatment for 24 h could significantly increase the intracellular ROS (Figures 2C, F), lipid peroxidation (Figures 2G–J) and Fe^2+^ contents (Figures 2K–N) of MG64 and 143B cells, which could be partially reversed by Fer-1 (10 uM). xCT and GPX4 is the key proteins of ferroptosis, Western blot results showed that shikonin (1.5 uM) treatment for 24 h could downregulate the expression of xCT and GPX4 proteins in OS cells (Figures 2O, P). Moreover, MDA is one of the important products of ferroptosis, so we subsequently detected the changes of MDA in MG63 and 143B cells. The results displayed that the MDA content increased in MG63 and 143B after shikonin (1.5 uM) treatment and partially reversed by Fer-1 (10 uM) (Figure 2Q). However, it was found that the GSH/GSSG ratio declined after shikonin (1.5 uM) treatment and partially reversed by Fer-1 (10 uM) in MG63 and 143B cells (Figure 2R). Taken together, our experimental results suggest that ferroptosis was involved in shikonin’s inhibition of the proliferation of MG63 and 143B cells.

### 3.3 Shikonin induced mitochondria damage in OS cells

**FIGURE 3:**
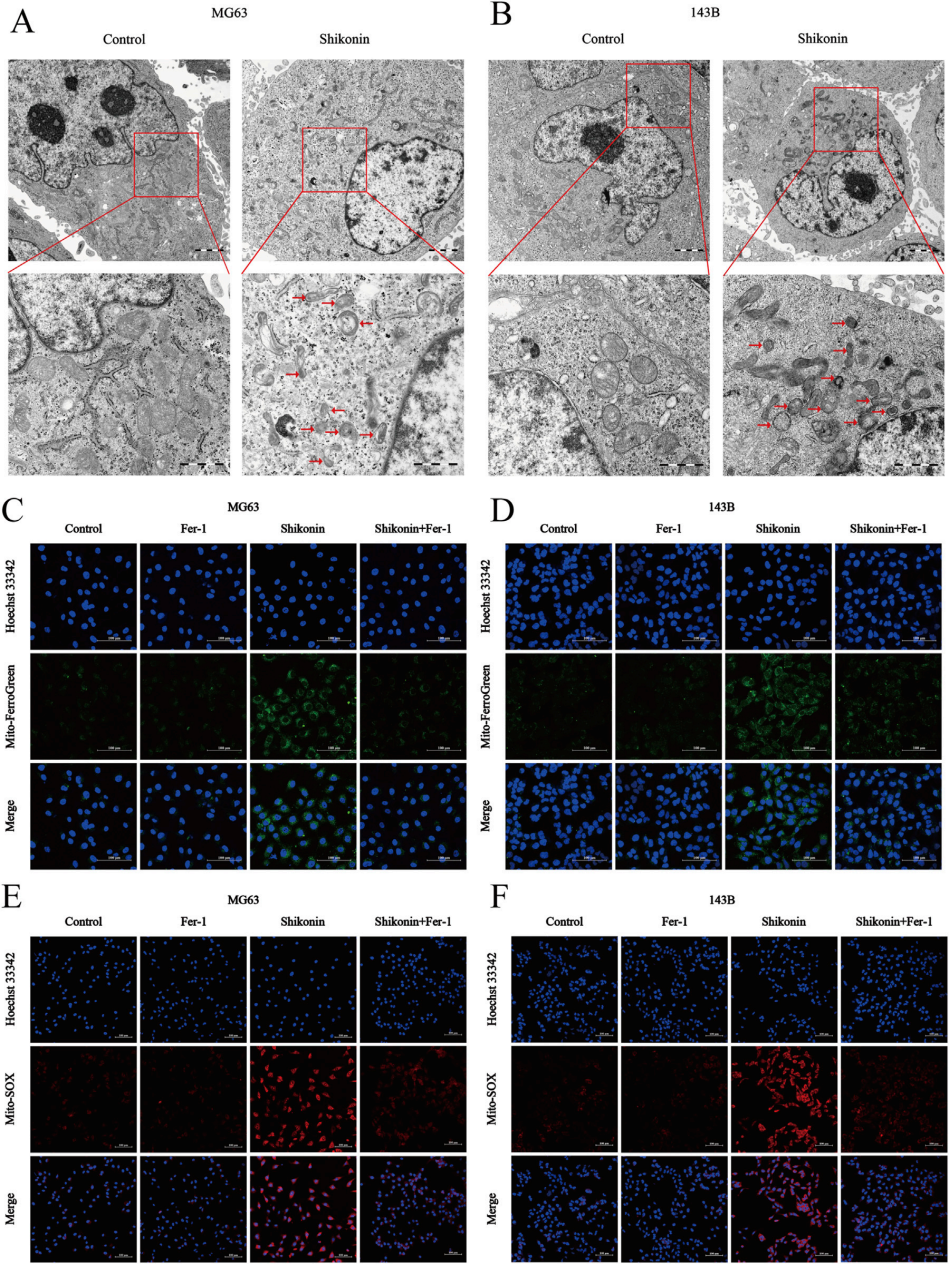
Shikonin induced mitochondria damage in OS cells. **(A, B)** The mitochondrial morphology of MG63 and 143B cells treated with or without shikonin after 24 h was observed by transmission electron microscopy (scale bar = 1 um). The red arrows represented mitochondrial changes typical of ferroptosis. **(C, D)** Mitochondrial Fe^2+^ and **(E, F)** mitochondrial ROS contents was observed by using Laser Scanning Confocal Microscopy (LSCM) after the intervention of co-treated with shikonin and Fer-1 after 24 h in MG63 and 143B cells (scale bar = 100 um).

Mitochondria, as the “energy pump” of cells, is one of the important organelles to maintain the normal metabolic activities of cells (Machihara et al., 2023). Transmission electron microscopy revealed that MG63 and 143B cells treated with shikonin (1.5 uM) after 24 h exhibited mitochondrial shrinkage, higher density of the mitochondrial membrane, and reduction or disappearance of mitochondrial cristae (red arrows), which are typical morphological features of ferroptosis, compared to control group (Figures 3A, B). Mitochondria are the core of redox homeostasis and participate in regulating iron homeostasis and cell metabolism during ferroptosis. Mito-FerroGreen was used to detect the changes of Fe^2+^ levels in mitochondria of MG63 and 143B cells after shikonin (1.5 uM) treatment for 24 h, and the results showed that shikonin could increase the level of mitochondrial Fe^2+^ (Figures 3C, D). Excessive Fe^2+^ in cells can produce a large number of ROS via the Fenton reaction. Mito-SOX fluorescent probe was used to detect mitochondrial ROS levels, and it was found that shikonin (1.5 uM) treatment for 24 h could also increase mitochondrial ROS in MG63 and 143B cells (Figures 3E, F).

### 3.4 Shikonin physically interacted with Nrf2 and promoted Nrf2 degradation

**FIGURE 4:**
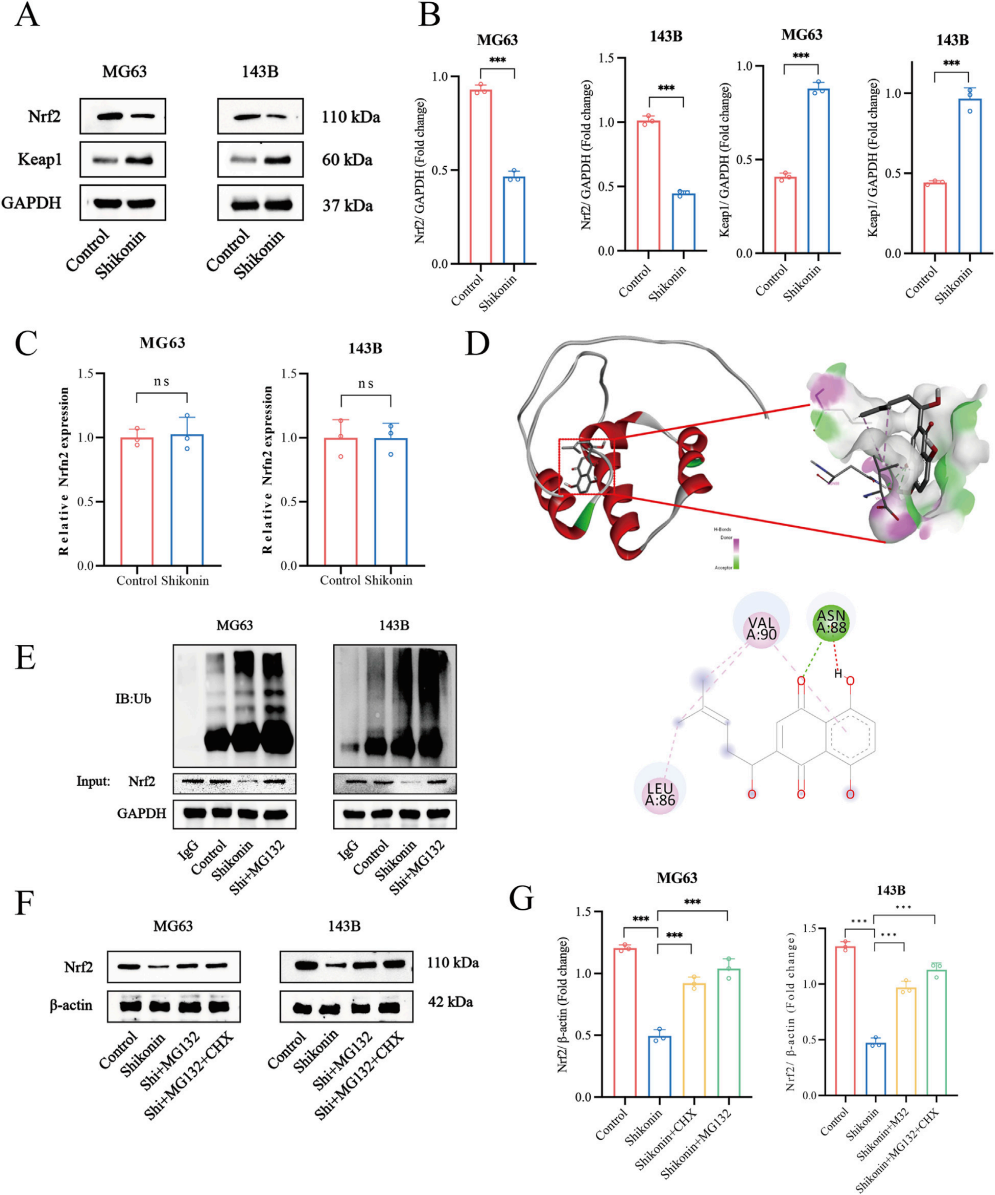
Shikonin physically interacted with Nrf2 and promoted Nrf2 degradation in OS cells. **(A, B)** Western blot was used to detect the expression of Nrf2 and keap1 in shikonin-treated MG63 and 143B cells, and quantification was analyzed. **(C)** Nrf2 mRNA levels in the control or shikonin (1.5 uM)-treated MG63 and 143B cells for 24 h. **(D)** The interaction between shikonin with Nrf2 was predicted by molecular docking. **(E)** Co-immunoprecipitation and Western blot assay of Nrf2 protein after shikonin and MG132 (2.5 uM) co-treatment 24 h in MG63 and 143B cells. **(F, G)** Western blot was used to observe the expression of the Nrf2 protein after the intervention of 1.5 uM shikonin cotreated with or without MG132 (2.5 uM) and CHX (35 uM) for 24 h, and quantification was analyzed. *p < 0.05, **p < 0.01, ***p < 0.001. The data were presented as mean ± SD, and analyzed using one-way ANOVA following Tukey’s t-test (n = 3).

Among the identified ferroptosis pathways, the xCT/GPX4 axis is the predominant negative regulatory system. Assessment of the expression of ferroptosis-related proteins showed that shikonin significantly suppressed the expression of xCT and GPX4. As a principal regulator of ferroptosis inhibitors xCT and GPX4, Nrf2 is normally kept at low levels through Kelch-like ECH-associating protein 1 (Keap1)-mediated ubiquitination and degradation (Guo et al., 2024). In this study, we found that shikonin (1.5 uM) significantly downregulated the expression of Nrf2 and upregulated the expression of Keap1 (Figures 4A, B). Interestingly, shikonin (1.5 uM) had no effect on Nrf2 mRNA levels (Figure 4C). Therefore, we speculated that shikonin suppressed Nrf2 expression through a post-transcriptional manner. Molecular docking was used to detect whether shikonin could directly interact with Nrf2 protein, and the results showed that shikonin may directly interact with Nrf2 to degrade it (binding energy of −5.8 kcal/mol) (Figure 4D). The ubiquitin-proteasome system is generally considered the most crucial protein degradation system under disuse conditions (Guo et al., 2022). We further utilized Nrf2 antibody to pull down endogenous Nrf2 proteins and examined their ubiquitin modification. Co-IP results showed that shikonin (1.5 uM) increased the endogenous ubiquitination of MG63 and 143B cells, and MG132 (2.5 uM) treatment increased the ubiquitin levels of Nrf2 (Figure 4E). CHX is a kind of inhibitor of protein synthesis. Under the action of CHX (35 uM), the degradation pathway of protein changes from the traditional ubiquitination dependent pathway to the non-ubiquitination dependent pathway. Western blot results showed that shikonin could reduce the expression of endogenous Nrf2, which could be reversed by MG132 and CHX to varying degrees (Figures 4F, G). These results suggested that shikonin might interact with Nrf2 proteins and contribute to the ubiquitination degradation of Nrf2, thereby inhibiting downstream xCT and GPX4 expression.

### 3.5 Overexpression of Nrf2 reversed the shikonin-induced ferroptosis

**FIGURE 5:**
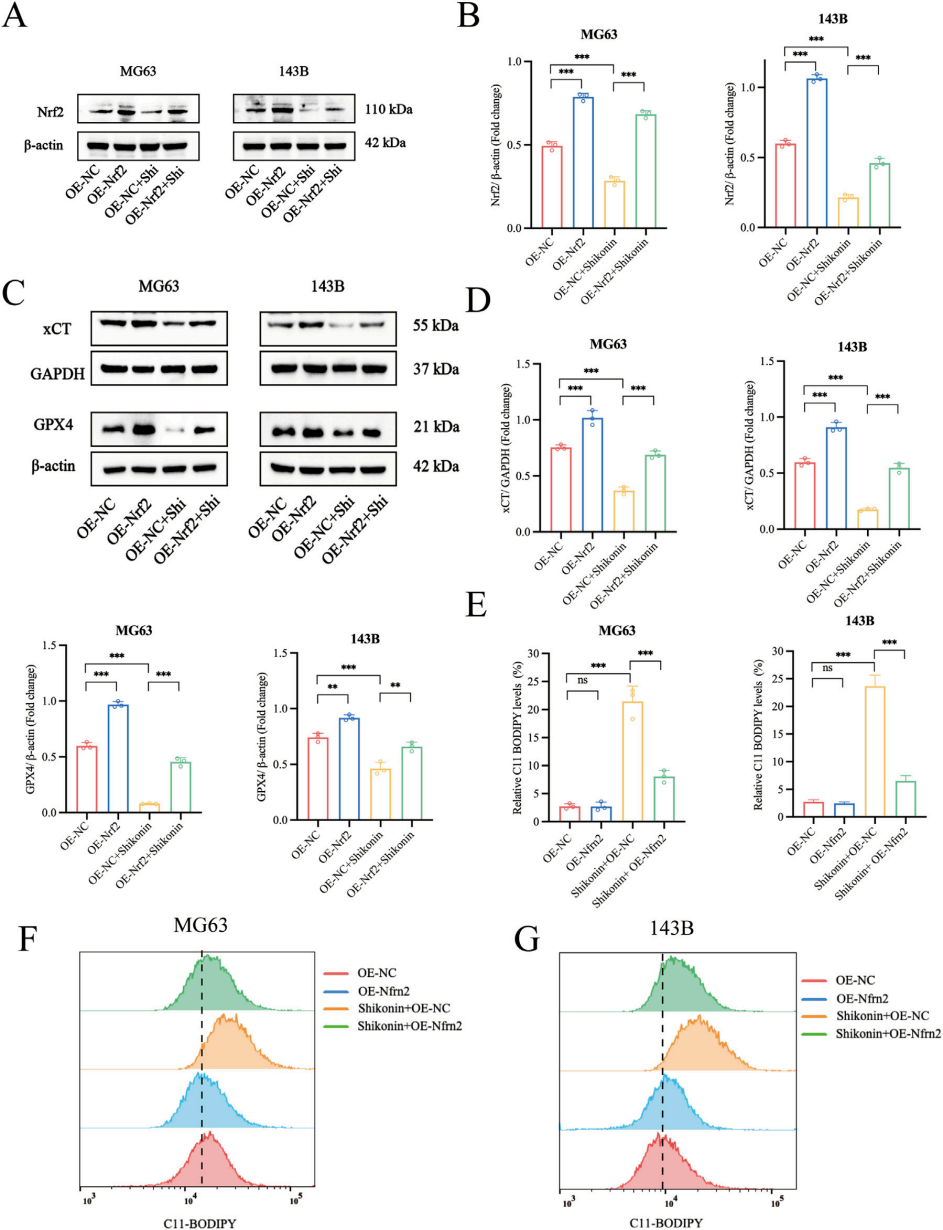
Overexpression of Nrf2 reversed the Shikonin-induced ferroptosis in OS cells. **(A–D)** Western blot was used to detect the expression of protein level of Nrf2, xCT and GPX4 in the shikonin treated Nrf2 overexpressing MG63 and 143B cells. **(E–G)** Flow cytometry was used to observe the total lipid peroxidation levels after the intervention of shikonin (1.5 uM) for 24 h in Nrf2 overexpressing MG63 and 143B cells. *p < 0.05, **p < 0.01, ***p < 0.001. The data were presented as mean ± SD, and analyzed using one-way ANOVA following Tukey’s t-test (n = 3).

Nrf2 is a specific regulator of antioxidant responses and plays a vital role in mitigating lipid peroxidation and ferroptosis. GPX4 and xCT are markers of ferroptosis. To further clarify the key role of Nrf2 signalling pathway is involved in shikonin-induced ferroptosis of MG63 and 143B, we established an Nrf2 overexpressing cell line (Figures 5A, B). As expected, overexpression of Nrf2 partially reversed the downregulation of xCT and GPX4 protein expression in MG63 and 143B cells induced by shikonin (1.5 uM) (Figures 5C, D). Lipid peroxidation is the critical step in the occurrence of ferroptosis. Similarly, overexpression of Nrf2 partially reversed the increase of lipid peroxidation in MG63 and 143B cells induced by shikonin (1.5 uM) (Figures 5E, G). These data suggested that Nrf2 overexpression rescued ferroptosis and phenotypic changes induced by shikonin.

## 4 Discussion

OS has been the most common primary bone malignant tumor in children and adolescents (Wang et al., 2020). The 5-year survival rate of OS without metastasis is about 65%, while that of patients with distant metastasis is only 19%–30% (Wang et al., 2022). Therefore, it is urgent to explore new therapeutic targets and to develop effective anti-OS drugs. More and more attention has been paid to the value of TCM extracts in the treatment of cancers (Xing et al., 2024). Shikonin is a kind of active ingredient extracted from comfrey, which has many pharmacological activities such as anti-inflammation and potent anti-cancer activities. However, imprecise mechanisms and targets greatly hinder its development as a candidate for anti-cancer drugs. In this study, we present significant evidence supporting the effective antitumor activity of shikonin against MG63 and 143B cells *in vitro*, significantly inhibited the rates of proliferation and markedly induced cell cycle arrest in dose-dependent manner. After confirming its anti-proliferation potential in OS cell line, the potential anti-tumor mechanism of shikonin was explored from the perspective of RCD subroutines. Notably, ferroptosis inhibitor have shown significant suppressive effects on the antitumor activity of shikonin, indicating that ferroptosis may be critical pathways for shikonin to treat OS.

As a novel mode of programmed cell death, ferroptosis is characterized by accumulation of iron, inhibition of GPX4 and increased lipid peroxidation (Kontoghiorghes et al., 2023). In addition, ferroptosis can be reversed by inhibitors Fer-1 and vitamin (El Hajj et al., 2023). The recent interest in determining the critical role of ferroptosis in malignancy inhibition is increasing (Wang et al., 2019). For example, bavachin induces ferroptosis via the STAT3/P53/SLC7A11 axis in OS cells (Luo et al., 2021). Chen et al. (2023) confirmed that the Anomanolide C inhibited the progression and metastasis of triple negative breast cancer by inducing autophagy-dependent ferroptosis via ubiquitinating GPX4. Thus, the induction of ferroptosis is a promising strategy for suppressing cancer cells progression. The present study showed that after treatment with shikonin, intracellular iron content, reactive oxygen species and lipid peroxidation levels increased, and these can be rescued by the ferroptosis inhibitor Fer-1. The morphological changes of the OS cells treated with shikonin were also consistent with ferroptosis, which mainly showed that the mitochondria became smaller, the membrane density increased, and the mitochondrial ridge decreased. These findings indicate that shikonin can induce iron death in OS cells.

Intracellular iron homeostasis is essential for cell survival, and the disturbance of intracellular iron metabolism, especially the increase of ferrous ion content, is the initial factor of ferroptosis. Moreover, when iron overload in tumor cells leads to excess ROS production through Fenton reaction, which promotes ferroptosis and tumor inhibition. Triggering lipid peroxidation, defined as the programmed process by which ROS strongly attack polyunsaturated fatty acids, is the canonical event that drives ferroptosis (Mo et al., 2023). MDA, the final product of lipid peroxidation, is an important end product of lipid ROS (Yin et al., 2022). Similarly, our results show that shikonin can increase the levels of ferrous ions, ROS, lipid peroxidation, and MDA in OS cells. These results strongly suggest that shikonin can induce ferroptosis in OS cells. Mitochondria are the “energy pumps” of cellular metabolic activities, and mitochondrial damage and morphological changes are usually the early events of cell death (Koenig et al., 2023). The most significant difference between ferroptosis and other types of programmed cell death is the change in mitochondrial (Wu Z. et al., 2023). Our further research results show that shikonin could increase mitochondrial Fe^2+^ level and ROS level, which declines the function of mitochondria. Morphologically, shikonin can cause mitochondrial volume reduction, membrane density increase, ridge reduction or disappearance, which is consistent with the mitochondrial characteristics of ferroptosis.

SLC7A11, the major component of system xCT, is mainly responsible for importing cystine, which is then reduced to cysteine and used to synthesize glutamate (Zheng et al., 2024). GPX4 oxidizes hydroperoxide and two molecules of reducing glutamate into lipid alcohols and oxidizing glutathione, acting as an antioxidant and free radical scavenger which can inhibit ferroptosis (Chen et al., 2022). GPX4 is a selenoprotein that belongs to the glutathione peroxidase family. The main function of GPX4 is to remove lipid peroxidation from the cell and prevent damage caused by peroxidation, which is essential for maintaining the integrity and function of cell membranes (Zhang and Xie, 2024). A previous study showed that shikonin can induce iron death in glioma cells by inhibiting xCT and GPX4 expression (Lu et al., 2018). We found that shikonin downregulates the expression of xCT and GPX4 and could be rescued by the ferroptosis inhibitor Fer-1 in OS cells, which is consistent with previous findings.

It is noteworthy that Nrf2 regulates ferroptosis through oxidative stress, and Nrf2 protects cells from ferroptosis by regulating the expression of xCT and GPX4, indicating that Nrf2 is a critical regulator of ferroptosis (Ge et al., 2021) Additionally, activation of the Nrf2/SLC7A11/GPX4 signaling pathway inhibits ferroptosis (Yuan et al., 2021). It was reported that the expression of Nrf2 in OS tissue was significantly higher than the normal peritumoral bone tissue, and it was closely associated with poor prognosis in OS patients (Zhang et al., 2016). Our data demonstrated that shikonin suppressed Nrf2 expression via inducing proteasomal degradation, and the proteasome inhibitor MG132 abrogated the suppressive effect of shikonin on Nrf2 expression. These data confirmed that shikonin induced ferroptosis in OS cells via facilitating Nrf2 ubiquitination and degradation. In general, Nrf2, as a transcription factor, which binds to its negative regulatory protein Keap1 and is ubiquitinated and degraded by the ubiquitin-proteasome system (Shuhua et al., 2022). Our further investigation indicated that Keap1 was obviously promoted by shikonin. These findings may shed light on the complicated molecular mechanisms of shikonin-induced cancer cell death, thereby opening a new perspective for plant lectins as potential anti-OS drugs in future cancer therapeutics. However, several limitations in this study should be noted. The *in vivo* anti-tumor effect of shikonin still remains to be verified, and relevant research should be carried out as soon as possible.

## 5 Conclusion

**FIGURE 6:**
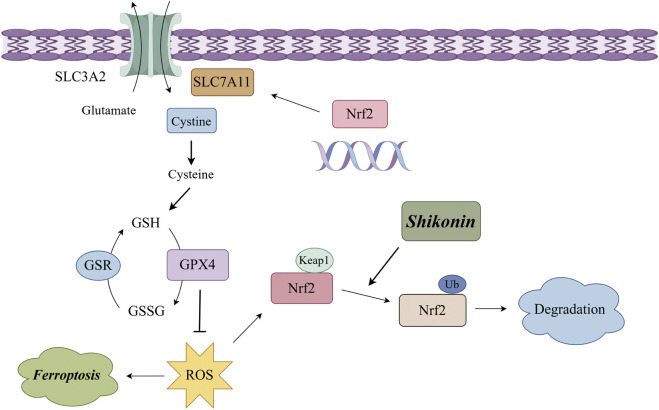
Schematic illustration of the molecular mechanism for shikonin induced ferroptosis of OS cells.

In conclusion, our findings supported that shikonin exerted anti-OS effects *in vitro* by triggering ferroptosis in OS cells. Mechanically, shikonin inhibits the expression of xCT and GPX4 by interacting with Nrf2 and promoting the ubiquitination degradation of Nrf2, thereby leading to ferroptosis (Figure 6). Hence, shikonin could be considered as a promising pharmaceutical for the treatment of OS.

## Data Availability

The original contributions presented in the study are included in the article/supplementary material, further inquiries can be directed to the corresponding author.

## Abbreviations

CHX, cycloheximide; Fer-1, ferrostatin-1; FDA, food and drug administration; MDA, malondialdehyde; RCD, regulated cell death; ROS, reactive oxygen species; OS, osteosarcomas; TCM, traditional Chinese medicine.
